# Impact of radiation on the incidence and management of ureteroenteric strictures: a contemporary single center analysis

**DOI:** 10.1186/s12894-021-00869-6

**Published:** 2021-08-04

**Authors:** Clinton T. Yeaman, Andrew Winkelman, Kimberly Maciolek, Mei Tuong, Perri Nelson, Chandler Morris, Stephen Culp, Sumit Isharwal, Tracey L. Krupski

**Affiliations:** 1 Department of Urology, UVA Medical Center, Fontaine Research Park, 500 Ray C. Hunt Drive, Charlottesville, VA 22908 USA; 2 University of Virginia School of Medicine, Charlottesville, VA USA

**Keywords:** Stricture, Ureteroenteric, Radiation, Urinary diversion

## Abstract

**Background:**

Ureteroenteric stricture incidence has been reported as high as 20% after urinary diversion. Many patients have undergone prior radiotherapy for prostate, urothelial, colorectal, or gynecologic malignancy. We sought to evaluate the differences between ureteroenteric stricture occurrence between patients who had radiation prior to urinary diversion and those who did not.

**Methods:**

An IRB-approved cystectomy database was utilized to identify ureteroenteric strictures among 215 patients who underwent urinary diversion at a single academic center between 2016 and 2020. Chart abstraction was conducted to determine the presence of confirmed stricture in these patients, defined as endoscopic diagnosis or definitive imaging findings. Strictures due to malignant ureteral recurrence were excluded (3 patients). Statistical analysis was performed using chi squared test, t-test, and Wilcoxon Rank-Sum Test, logistic regression, and Kaplan–Meier analysis of stricture by cancer type.

**Results:**

65 patients had radiation prior to urinary diversion; 150 patients did not have a history of radiation therapy. Benign ureteroenteric stricture rate was 5.3% (8/150) in the non-radiated cohort and 23% (15/65) in the radiated cohort (*p* =  < 0.001). Initial management of stricture was percutaneous nephrostomy (PCN) in 78% (18/23) and the remaining 22% (5/23) were managed with primary retrograde ureteral stent placement. Long term management included ureteral reimplantation in 30.4% (7/23).

**Conclusions:**

Our study demonstrates a significant increase in rate of ureteroenteric strictures in radiated patients as compared to non-radiated patients. The insult of radiation on the ureteral microvascular supply is likely implicated in the cause of these strictures. Further study is needed to optimize surgical approach such as utilization of fluorescence angiography for open and robotic approaches.

## Background

Ureteroenteric anastomotic stricture is a well-known complication of urinary diversion which occurs in 4–20% of patients [1–3]. Patients with strictures present clinically with flank pain, decreased renal function, pyelonephritis, or may be asymptomatic and diagnosed incidentally with progressive hydronephrosis and renal impairment. Strictures are a cause of significant morbidity and health care expenditure, as treatment necessitates procedural intervention and often several repeat admissions. Numerous studies have sought to identify risk factors for development of ureteroenteric strictures, as well as opportunities for technical approaches to reduce stricture risk. Stricture is traditionally more common on the left side, in patients with elevated BMI, longer operative time, and those with Clavien-Dindo ≥ 3 complication, however these studies have not identified radiation therapy as a significant risk factor [3–6]. Increased stricture risk of the left side has been attributed to the need for greater ureteral mobilization in order to transpose it to the right side for anastomosis. This extended dissection is likely to compromise distal ureteral vasculature. Operative time and high-grade complications are associated with stricture development likely in part due to fluid shifts, hypoperfusion of the ureteroenteric anastomosis. These as well as increased risk with elevated BMI are suggestive of the risks seen with difficult urinary diversion surgeries.

Ischemia of the anastomosis related to the tenuous ureteral blood supply is implicated in the formation of ureteroenteric stricture [7]. Basic science and animal data have established the detrimental impact of radiation therapy on microvasculature [8]. Radiation-induced ureteral ischemia, in conjunction with periureteral fibrosis, make the preservation of distal ureteral blood supply during urinary diversion tenuous. This is of concern in patients requiring pelvic exenteration for colorectal or gynecological malignancy, which may necessitate radiation therapy prior to surgical intervention [9–11]. Radiation history is also an important contributor commonly in patients undergoing benign diversion for radiation cystitis or fistulas. Radiation has been demonstrated to be an independent risk factor for development of ureteral strictures, with a 2% ureteral stricture rate from radiation therapy alone [11]. One institutional review examined 25 ureteral strictures and found urolithiasis was the most common cause (60%) while 28% were due to radiation, 4% endometriosis, 4% iatrogenic (without stone), and 4% unknown or other causes [12]. These findings were demonstrated in a cohort of patients with intact urinary tracts and have not been reproduced in a cohort undergoing urinary diversion. Recent work by Beller et al. demonstrated that patients with ureteral stricture following radiation therapy for gynecologic malignancy underwent a mean of 25 procedures per patient, highlighting the significant morbidity and cost associated with ureteral stricture [13].

Radiation therapy continues to improve targeting techniques that spare adjacent organs and urologists have more precision with robotic assisted techniques. With these new innovations, it is possible radiation is less of a risk factor than previously thought. We sought to determine if radiation continues to adversely affect ureteroenteric anastomotic stricture rates in a contemporary series and identify other contributing risk factors.

## Methods

An IRB-approved prospectively maintained cystectomy database was utilized to identify ureteroenteric strictures amongst patients who underwent urinary diversion at a single academic center between 2016 and 2020. The cohort of interest was any patient who underwent a urinary diversion whether it was for a benign and malignant etiology. Particular interest was in those with prior radiotherapy for colorectal, gynecologic or urologic malignancy. There were no exclusions in terms of surgeon, operative technique, or type of diversion. Even for robotic extirpation, the diversion was performed in open fashion. For robotic-assisted cases, the ureteral dissection was completed robotically. All ureteroenteric anastomoses were completed using the Bricker technique with interrupted absorbable monofilament. The most common suture used in anastomosis was 4–0 monocryl. Postoperative care was conducted per surgeon preference, including length of stent duration. Procedurally, care was taken to limit ureteral mobilization and manipulation and preserve periureteral tissue.

The outcome of interest was ureteroenteric anastomotic stricture. Chart abstraction was necessary to evaluate imaging reports and clinical presentation to confirm the presence of a clinically relevant stricture. We defined a stricture as endoscopic diagnosis or definitive imaging findings. Endoscopic diagnosis was defined as visualization of focal narrowing at the anastomosis on ureteroscopy, and definitive imaging findings were defined on Computed Tomography (CT) reports indicating focal narrowing at the anastomoses with related hydronephrosis. In the latter case, patients were excluded from the confirmed stricture cohort if their ureteral stricture was suspected to be a result of malignant recurrence; such findings would be indicated by the presence of focal enhancement at the site of ureteral obstruction/stricture and/or biopsy pathology with proven malignancy. Radiation dose delivered was extrapolated based upon current cancer guideline-based protocols. Statistical analysis performed using chi squared test, Student’s T-test, Wilcoxon Rank-Sum Test, and logistic regression where appropriate using α = 0.05. Given the heterogeneity of the cohort, subset analysis was performed comparing stricture rates between the highest volume surgeon and the remaining surgeons for both radiated and non-radiated patient populations using chi square test. A Kaplan–Meier survival curve was used to compare UES rates between radiation treated cancers.

## Results

**Fig. 1:**
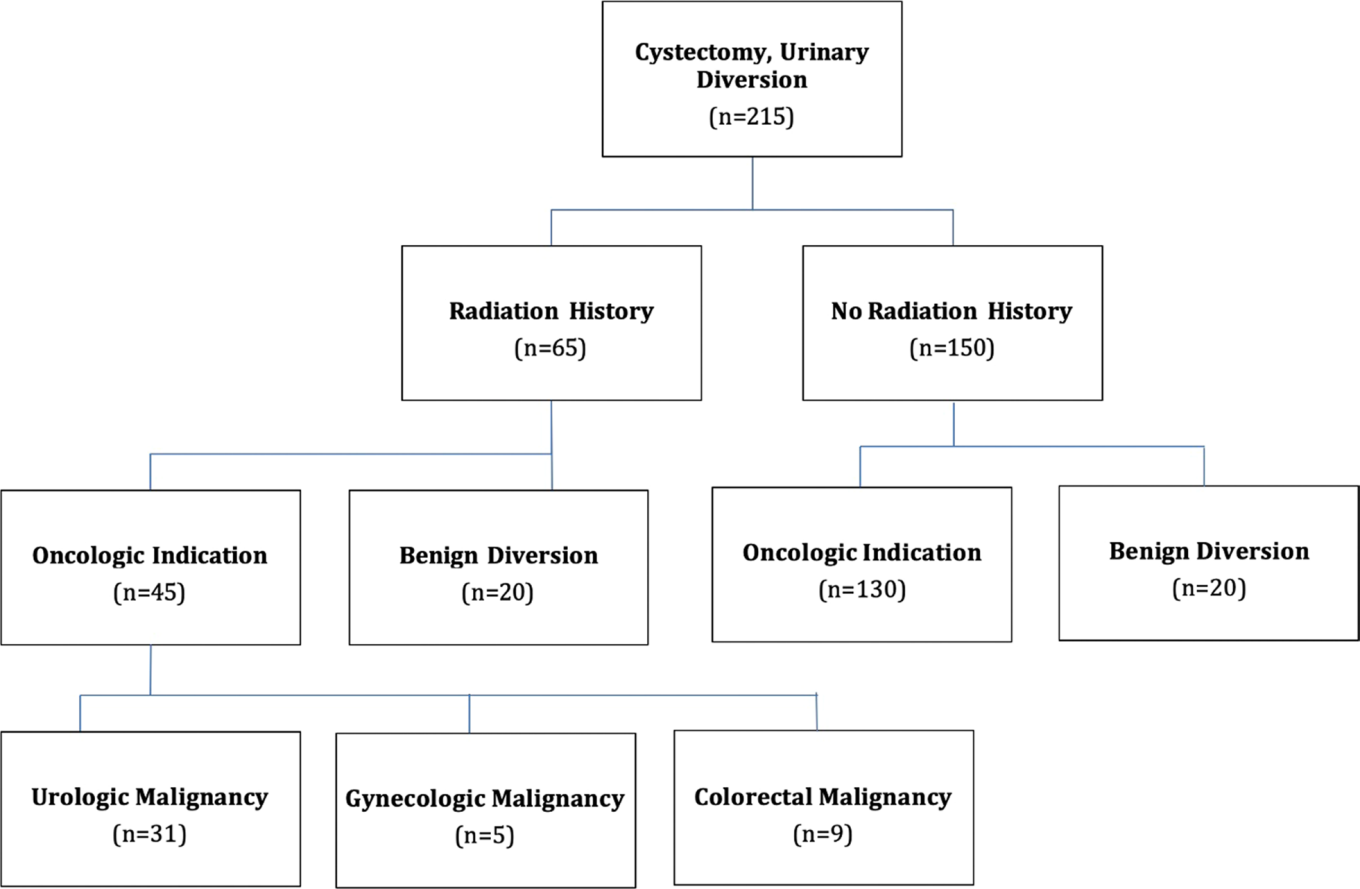
Breakdown of patient cohorts, radiation status, and indication for urinary diversion

Over the four year period ending Feb 2020, 215 patients with urinary diversions performed by six surgeons were identified. Figure 1 shows the total cohort broken down by benign versus malignant etiology and for those with malignancy, the number with a non-urologic malignancy. 196 patients underwent construction of ileal conduit, while the other 19 had continent diversions, all of which had refluxing Bricker ureteroenteric anastomoses. 65 patients underwent radiation prior to urinary diversion; 150 patients did not undergo preoperative radiation. The demographics of the cohort comparing the radiated to non-radiated patients are shown in Table 1, including operative intervention and chosen urinary diversion. Patients who underwent pelvic exenteration and supratrigonal cystectomy were more likely to have had radiation, while patients who had radical cystectomy and urinary diversion (RCUD) were less likely to have had radiation (p < 0.0001). Patients who underwent radiation therapy were more likely to undergo ileal conduit rather than continent diversion (*p* = 0.013). There was no significant difference between age, sex, Body Mass Index (BMI), Charlson Comorbidity Index, or robotic-assisted surgical approach. The length of stent duration postoperatively was a median of 6 days for the non-radiated group and 7.5 days for the radiated group (*p* = 0.08). The median stent duration postoperatively for patients who would go on to develop stricture was 5.5 days in the non-radiated group and 5 days in the radiated group. In the non-radiated cohort, 7 patients did not have surgical excision of their primary pathology, but did undergo urinary diversion. In the radiated cohort, 10 patients did not have surgical excision of their primary pathology. There was no association between primary pathologic resection and ureteroenteric stricture development.

**Table 1:** Demographic and surgical information

	Overall cohort (n = 215)	Non-radiated (n = 150)	Radiated (n = 65)	*p*
	N (%)	N (%)	N (%)	
Age	66.5	65.3	67	0.2
BMI (kg/m^2^)	27.57	28.93	26.98	0.06
Female	70 (32.5)	44 (29.3)	26 (40.0)	0.12
CCI	3.9	3.6	4.1	0.1
Pelvic exenteration	15 (7)	0	15 (23.1)	< 0.0001
				< 0.0001
Radical cystectomy/urinary diversion	160 (74.4)	130 (86.6)	30 (46.2)	0.002
Supratrigonal cystectomy/urinary diversion	40 (18.6)	20 (13.3)	20 (30.8)	
Ileal conduit	196 (91)	132 (88)	64 (98.5)	0.01
Orthotopic neobladder	7 (3)	7 (4.7)	0	
Indiana pouch	12 (6)	11 (7.3)	1 (15.3)	
Robotic-assisted	22 (11)	18 (12)	4 (6.2)	0.19
Open approach	193 (89)	132 (88)	61 (93.8)	

BMI, body mass index; CCI, Charlson comorbidity index

The overall benign ureteroenteric stricture rate was 10.6% (23/215). Ureteroenteric stricture rate was 5.3% (8/150) in the non-radiated cohort and 23% (15/65) in the radiated cohort (p < 0.0001). Stricture diagnosis was made on average 10.3 months after urinary diversion. Logistic regression was used to adjust for BMI as a risk factor. Radiation was found to be a significant predictor of ureteroenteric stricture (*p* = 0.0005) while BMI was not significant (*p* = 0.75). The odds ratio for radiation was 5.2. Diagnosis was made on average 12.7 months postoperatively in radiated patients and 5.7 months postoperatively in non-radiated patients, however this difference was not significant (*p* = 0.06). Of those with prior radiation, 10 were left-sided, 4 were right-sided, and 1 was bilateral. In the non-radiated group, 5 were right-sided and 3 were left-sided. Initial management of stricture was percutaneous nephrostomy (PCN) in 78% (18/23) and the other 22% (5/23) were managed with primary ureteral stent. 30.4% (7/23) patients went on to have open reimplantation, 52.2% (12/23) continued to have stent exchanges, and 17.4% (4/23) live with percutaneous nephrostomy tubes. 3 of the 7 patients who had open reconstruction had prior radiation and 4 were non-radiated.

There was no significant difference between rates of 90-day readmission. To account for variability in stricture rates between surgeons, we analyzed rates of stricture between the single surgeon who performed 127 of the included cases in comparison with the 5 surgeons who performed the remaining 88 cases. There was no difference in stricture risk by surgeon for those with radiation (*p* = 0.2) or those without radiation (*p* = 0.07). Clinical presentation of each of the ureteroenteric strictures was determined. Each of these clinical factors was not mutually exclusive. Pyelonephritis was the most common presenting symptom of stricture. 6 non-radiated and 8 radiated patients presented with pyelonephritis. 2 non-radiated patients and 8 radiated patients presented with renal impairment. 1 non-radiated patient and 3 radiated patients experienced flank pain. 3 radiated patients were clinically asymptomatic and diagnosed on imaging. Of the 3 patients who were diagnosed primarily with hydronephrosis noted on imaging, 2 had renal impairment noted on concurrent laboratory evaluation as well. Of note, nine patients would go on to have ureteroscopy confirming stricture presence, however each of the diagnosed strictures was first evaluated with cross-sectional imaging, which demonstrated the presence of ureteroenteric stricture. Six patients had a Mag-3 renal lasix scan performed as a component of their work-up for stricture.

**Fig. 2:**
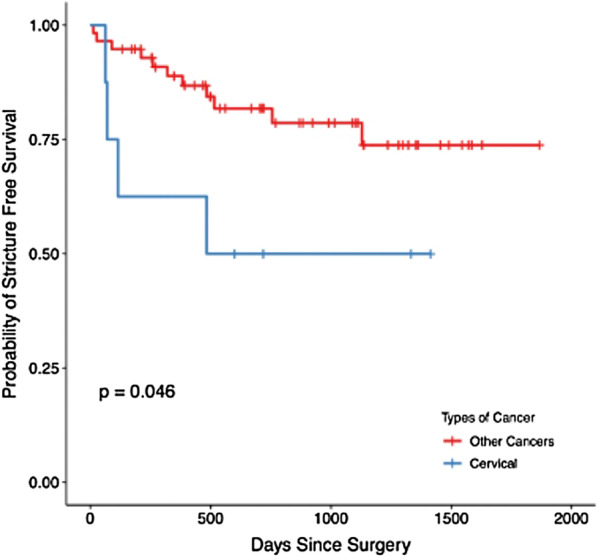
Ureteroenteric stricture free survival by radiation treated cancers

Within the radiated cohort, we examined patients by indication for radiation therapy. 7 patients underwent radiation for cervical cancer (target dose 85 Gy) and 4 patients (57%) experienced ureteroenteric stricture. 11 patients had radiation for colorectal malignancy (target dose 50 Gy) and 3 patients (27%) developed strictures. 40 patients had radiation therapy for urologic malignancy (target dose 45–50 Gy pelvis), with 7 patients (18%) developing ureteroenteric stricture. An additional 7 patients had radiation therapy for other non-cervical gynecologic malignancies (45–50 Gy) and 1 patient experienced ureteroenteric stricture. Kaplan–Meier estimation demonstrated radiation treated cervical cancer had a statistically significant higher probability of developing UES compared with other radiation treated cancers (*p* = 0.046), which is shown in Fig. 2.

## Discussion

Our study demonstrates evidence that a contemporary series of patients with a history of prior radiation therapy had higher rates of ureteroenteric stricture following urinary diversion while other covariates were similar with an odds ratio of 5.2. This is in spite of our knowledge of radiation as a risk factor and utilizing known strategies for mitigation such as extensive spatulation, mucosal eversion and no-touch technique. It is also the practice of our surgeons, including the highest volume surgeon, to emphasize only mobilization and manipulation of the ureters to the degree surgically necessary and preservation of ureteral vasculature is paramount. Though previous studies have identified BMI as a risk factor for stricture, we did not find this to be significant in our series. Ureteroenteric stricture remains a well-known complication of urinary diversion, yet little progress has been made in reduction of stricture rates, either by means of surgical technique or postoperative management [5, 6, 14–17]. Demonstrating that radiation is a risk factor for ureteroenteric stricture is important in preoperative counseling, particularly for exenteration, continent or incontinent diversion selection, and possibly post-operative imaging follow up. It is well demonstrated that radiation therapy affects tissue causing fibrosis and impairment of microvascular tissue perfusion. Ischemia to the distal ureter due to compromise of the microvascular blood supply is implicated in the formation of ureteroenteric strictures [7].

Several groups have tested whether varied surgical methods may offer risk reduction. Surgical anastomotic approach has not significantly impacted ureteroenteric stricture rate [5, 14–16]. However, a more recent study suggests that intracorporeal diversion confers a higher risk of ureteroenteric stricture [14]. This is counterintuitive as robotics is advertised as more precise with better visualization. Later work has suggested that this increased risk noted with intracorporeal diversion diminishes after a surgeon performs 75 diversions on the learning curve [18]. A study of 47 robotic assisted urinary diversions by Ahmadi et al. demonstrated intraoperative use of indocyanine green had a lower ureteroenteric stricture rate [19]. Recent work by Shen et al. has demonstrated potential for the use of intraoperative fluorescent angiography during open urinary diversion in reduction of ureteroenteric stricture rates. In a series of 47 patients, no ureteroenteric strictures were detected in the experimental group [20]. These studies show promise for widespread reduction in ureteroenteric stricture rates by assessing ureteral perfusion prior to making ureteroenteric anastomosis, as the technique will not be limited to robotic only approach. The gold standard for treatment of ureteroenteric stricture is open repair with ureteral reimplantation. These are challenging surgeries and are most successful when redundancy in the conduit allows the bowel to be moved to the non-diseased ureter. Patients must be counseled on the risk of nephrectomy or continued stricture if reimplantation fails. Open surgical repair has a success rate of 92–100%, while endoscopic management has a success rate of 30–50% [21–23].

We found that patients who underwent pelvic exenteration and supratrigonal cystectomy were more likely to have had radiation. The difference within the pelvic exenteration population is attributable to the standard of care for gynecologic and colorectal malignancy consisting of neoadjuvant radiation therapy protocols [9–11]. Obtaining exact radiation dosing information by chart abstraction proved difficult as many patients undergo radiation therapy in the community outside of our institution. We did note a high incidence of ureteroenteric stricture in those who had radiation for cervical cancer, however this study was not powered to evaluate differences on the basis of malignancy type. Cervical cancer has a high radiation dose (85 Gy) delivered in close proximity to the distal ureters [9]. Additionally, there exists the risk of iatrogenic ureteral devascularization in those patients with masses abutting or invading the ureters. This is of particular interest in the radiated gynecologic malignancy patient, where radiation may impair the development of collateral blood supply in the setting of iatrogenic ureteral vascular compromise. For some patients who underwent supratrigonal cystectomy, the indication was radiation cystitis or fistula, which contributes to that demographic difference. We found that while the stricture rate was higher within the radiation cohort, the 90-day readmission rate was not significantly higher. While cystectomy and urinary diversion remains a complex surgery that carries high risk of readmission, lowering rates of ureteroenteric stricture would alleviate the morbidity associated with repeated procedural intervention as well as readmission beyond 90 days. With mean time to stricture diagnosis of 10.3 months postoperatively, reduction in readmission in the early postoperative period may not be seen, but reduction in late readmission and concomitant costs may be realized. Ureteroenteric stricture and related complications and readmissions also confer significant healthcare cost and remain one of the most costly per day reasons for readmission following urinary diversion [24]. Treatment of a stricture with prolonged stent placement also obviates a risk factor for the rare but catastrophic ureteroiliac fistula.

A factor that makes study and minimization of ureteroenteric stricture difficult is the lack of consensus on stricture definition and proper diagnosis. We defined stricture as endoscopic diagnosis or definitive imaging findings. Endoscopic diagnosis consisting of visualization of focal narrowing at the anastomosis on ureteroscopy, and definitive imaging findings on CT indicating focal narrowing at the anastomoses with related hydronephrosis without concern for malignant recurrence. This is consistent with work by other groups [24]. However, others have defined ureteroenteric stricture by the need for future procedures [25]. Further, urologists have different protocols for postoperative surveillance imaging which may detect asymptomatic/subclinical ureteroenteric strictures. The varied utilization of cross-sectional imaging, loopogram, antegrade/retrograde fluoroscopy, Mag-3 renal lasix scan, and ureteroscopy in the diagnosis of ureteroenteric stricture offers opportunity for standardization of the clinical workup. With the workforce shortage in urology, we often have no local urologists to perform follow-up testing. It is possible our surveillance is closer, leading to detection bias. Our colorectal and gynecologic surgeons prefer to perform total pelvic exenterations in an open fashion as opposed to robotic assisted. Further, our institution serves a predominately rural population and patients often presents with advances disease at initial presentation, which necessitates a larger number of open cystectomies.

Ureteroenteric stricture minimization remains an area of need for continued research. The use of fluorescent angiography or indocyanine green and firefly technology gives surgeons the ability to assess ureteral perfusion and have demonstrated significant improvement in ureteroenteric stricture rates in single institution series [19, 20]. Further work is merited to validate these findings and incorporate them into standard practice.

## Conclusion

Our study demonstrates a significant increase ureteroenteric strictures rate in radiated patients as compared to non-radiated patients. The insult of radiation on the ureteral microvascular supply is likely implicated in the cause of these strictures. Further study is needed to optimize surgical approach such as utilization of indocyanine green fluorescence angiography or fluorescein angiography.

## Data Availability

The datasets used and/or analyzed during the current study are available from the corresponding author on reasonable request.
